# The impact of recent chemotherapy on immunity in 2 COVID-19 cases with gastrointestinal tumors: A case report

**DOI:** 10.1097/MD.0000000000026143

**Published:** 2021-05-28

**Authors:** Zhaoqing Ju, Jun Yang, Gang Lu, Jian Li, Yun Wu, Xiaofeng Wu, Yanjie Huang, Yi Ai, Dongfeng Xiang, Bo Zeng, Zuwang Yang, Nianqiao Gong

**Affiliations:** a The Enshi Center for Disease Control and Prevention, The Health Committee; b The Central Hospital of Enshi Autonomous Prefecture, Enshi; c Institute of Organ Transplantation, Tongji Hospital, Tongji Medical College, Huazhong University of Science and Technology; d Key Laboratory of Organ Transplantation, Ministry of Education, NHC Key Laboratory of Organ Transplantation, Key Laboratory of Organ Transplantation, Chinese Academy of Medical Sciences; e Hepatic Surgery Center, Tongji Hospital, Tongji Medical College, Huazhong University of Science and Technology, Wuhan; f Internal Department, People's Hospital of Lichuang; g Infectious Department, People's Hospital of Xianfeng, Enshi, China.

**Keywords:** carcinoma, chemotherapy, coronavirus disease 2019, gastrointestinal tumors, immune function

## Abstract

**Introduction:**

Coronavirus disease 2019 (COVID-19) is a rapidly emerging infectious respiratory disease caused by severe acute respiratory syndrome coronavirus 2. Currently, more than 100 million cases of COVID-19 have been confirmed worldwide, with over 2.4 million mortalities. The pandemic affects people of all ages but older individuals and those with severe chronic illnesses, including cancer patients, are at higher risk.

**Patient concerns:**

The impact of cancer treatment on the progression of COVID-19 is unclear. Therefore, we assessed the effects of chemotherapy on COVID-19 outcomes for 2 cancer patients. On January 24, 2020, a level I response to a major public health emergency was initiated in Hubei Province, China, which includes Enshi Autonomous Prefecture that has a population of 4.026 million people. As of April 30, 2020, 252 confirmed cases of COVID-19 and 11 asymptomatic carriers were identified in Enshi.

**Diagnosis:**

Among the confirmed cases and asymptomatic carriers, 2 patients were identified who were previously diagnosed with malignant tumors, including one with hepatocellular carcinoma and the other with cardia carcinoma.

**Interventions:**

These 2 patients were receiving or just completed chemotherapy at the time of their COVID-19 diagnosis.

**Outcomes:**

Both patients were followed and presented favorable outcomes. The positive outcomes for these 2 patients could be partially explained by their recent chemotherapy that impacted their immune status. Also, their relatively younger ages and lack of comorbidities were likely factors in their successful recovery from COVID-19.

**Conclusions:**

Anticancer treatment might enhance a patient's ability to respond favorably to COVID-19 infection. However, anticancer treatment is likely to impact immune function differently in different individuals, which can influence disease outcomes.

## 1 Introduction

On January 24, 2020, the first confirmed case of coronavirus disease 2019 (COVID-19) was verified in Enshi, and a level I major public health emergency response was initiated. The multifaceted public health interventions resulted in no additional confirmed cases after February 28, 2020, 36 days after the first confirmed case of COVID-19. The morbidity rate, based on the whole population including confirmed cases and known asymptomatic carriers, was 0.00653% (263/4.0261 million). Therefore, public health intervention measures played a pivotal role in limiting the spread of COVID-19 in Enshi. In Wuhan, the implementation of public health interventions also was associated with a reduction in the reproduction number (Rt) from a peak of 3.82 to below 1.0 on February 6, 2020, and below 0.3 on March 1, 2020.^[1]^

As of April 30, 2020, 252 confirmed cases and 11 asymptomatic carriers were confirmed in Enshi. Seven individuals who died were verified to have been infected with COVID-19. The rapid and effective medical treatment resulted in a relatively low mortality rate of 2.78% for confirmed COVID-19 cases (7/252). This result was different from what was observed in the early stage of the Wuhan outbreak, which exhibited rapid overcrowding of available medical facilities. The appropriate public health strategy and timely medical treatment ensured that Enshi experienced adequate control of the COVID-19 outbreak. After February 22, 2020, no more confirmed cases of COVID-19 have been reported in Enshi. On March 17, 2020, the last confirmed COVID-19 case was discharged from the hospital designated to treat patients diagnosed with COVID-19.

Currently, the number of confirmed cases worldwide has surpassed 100 million, with more than 2.4 million deaths due to COVID-19. The pandemic has involved all parts of the global population, including cancer patients. However, the impact of cancer treatment on the progression of COVID-19 infections remains unclear. Herein, we introduced the possible beneficial effects of chemotherapy on COVID-19 outcomes for cancer patients. Among the confirmed cases and asymptomatic carriers in Enshi, 2 patients presented with malignant tumors (1 hepatocellular carcinoma and 1 cardia carcinoma) and were receiving or just completed chemotherapy at the time of their COVID-19 diagnosis. Both patients were followed and included in this case report.

## 2 Case report

### 2.1 Case 1

**Figure 1:**
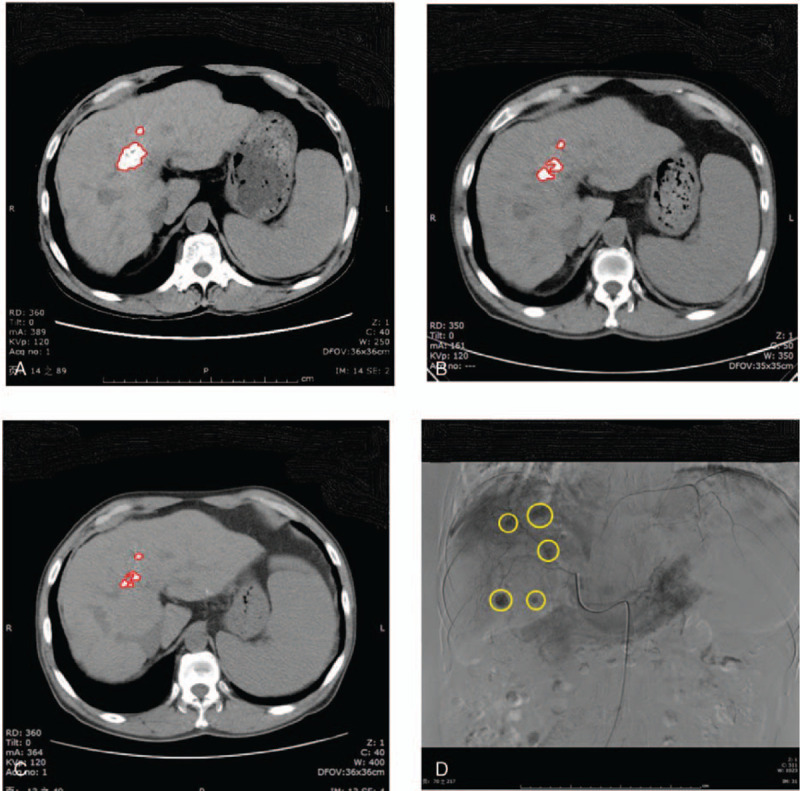
Liver images from case 1 A. After the first TACE (December 18, 2018), the liver plain CT scan showed that the liver had multiple high-density shadows. B. After the second TACE (March 19, 2019), the liver plain CT scan showed that some of the high-density shadows in the liver were smaller than before (December 18, 2018). C. After the third TACE (November 27, 2019), the liver plain CT scan showed that some of the high-density shadows in the liver were smaller than before (March 19, 2019); D. The fourth TACE (November 29, 2019) angiography showed the superior deposition of lipiodols. The circles indicate the tumors.

The first case was a 55-year-old male without a history of chronic disease but had been diagnosed with liver cancer more than 1 year previously. This patient underwent 4 transcatheter arterial chemoembolizations (TACE) in Wuhan on November 16, 2018, and January 18, March 22, and November 29, 2019. The Seldinger method was used each time for the local injection of the chemotherapy drug (50 mg of lobaplatin) (Fig. 1). Starting on December 2, 2019, the patient took a targeted oral drug for liver cancer (sorafenib 0.4 g bid) until February 9, 2020.

On January 27, 2020, the patient experienced a sudden fever (38.8°C), chills, cough, and expectoration that was accompanied by shortness of breath after physical activity. The fever and cough were not relieved after taking ibuprofen at home. On January 28, 2020, the patient went to the hospital and was admitted the same day (Day 0, D0). A routine blood examination revealed the following parameters, leukocytes at 6.64 × 10^9^/L, lymphocytes at 0.79  × 10^9^/L, and C-reactive protein at 104.28 mg/L. Tests for influenza A virus antigen and the respiratory 9 joint examination were negative. Viral pneumonia was diagnosed based on a chest computed tomography (CT) scan. On D2, a routine blood test revealed that his leukocyte count was 5.88 × 10^9^/L, and lymphocyte count was 0.78 × 10^9^/L. The chest CT scan showed that both of the patient's lungs were severely infected. The patient exhibited wheezing and dyspnea at 22:36 hours and was immediately transferred to the intensive care unit. Tracheal intubation was performed, and continuous ventilator-assisted respiration was initiated. While the patient was in the intensive care unit, he received the following treatments, anti-infection, hormone shock, anti-inflammation, antivirus, electrolyte supplementation, and maintenance of body fluids.

**Figure 2:**
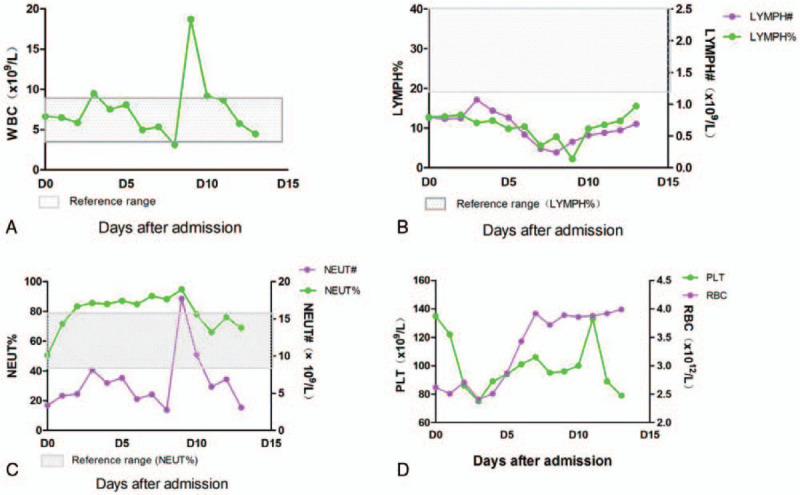
The changes in blood cells during the hospitalization of case 1. A. The leukocyte number. B. The lymphocyte ratio and number. C. The neutrophil ratio and number. D. The platelet count and red blood cell number. The shadows represent the normal ranges for the parameters.

**Figure 3:**
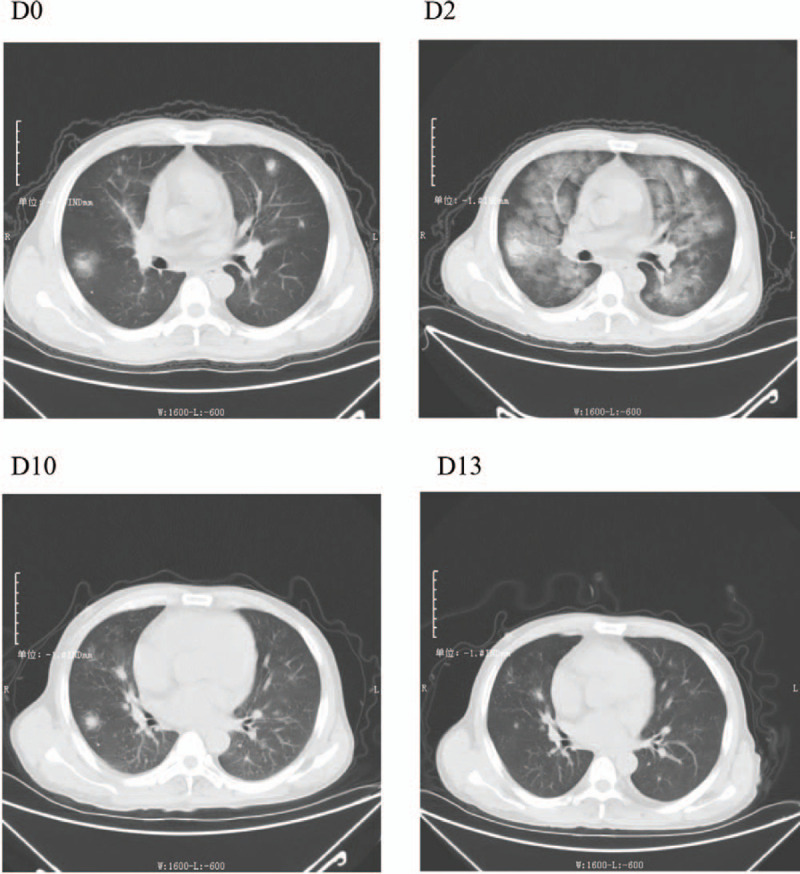
The changes in the lung plain CT scan of case 1 throughout treatment. D0. Bilateral lung inflammation. D2. Bilateral lung lesions were advanced. D10. Bilateral lung lesions were decreased. D13. The lesion range was reduced, and the density was light.

On D4, a polymerase chain reaction (PCR) nucleic acid test (Shanghai Berger Technology Co., Ltd.) on a nasopharyngeal swab specimen from this patient was positive for severe acute respiratory syndrome coronavirus 2 (SARS-CoV-2). The patient was confirmed as having COVID-19 pneumonia (critical type). On D6, his condition improved and the endotracheal tube was removed. On D10, a chest CT scan showed that the lung lesions had continued to be absorbed. On D11, the patient had recovered to a mild disease status based on his arterial blood gas analysis. His oxygenation index was 227 mm Hg, and he did not exhibit any signs of respiratory failure. On D13, a chest CT scan showed that the lung infection was markedly reduced compared with the previous images. The patient's respiratory rate was less than 30 times per minute, his oxygen saturation was maintained above 95% at rest, and his oxygenation index was maintained above 300 mm Hg. On D15, the patient's body temperature had been normal for more than 3 days, and his respiratory symptoms were significantly improved. A routine blood test performed on D15 is seen in Fig. 2. A chest CT scan demonstrated that the pulmonary lesions were significantly reduced (Fig. 3). Two consecutive (D12, D14) SARS-CoV-2 nucleic acid tests from nasopharyngeal swab specimens were negative.

**Figure 4:**
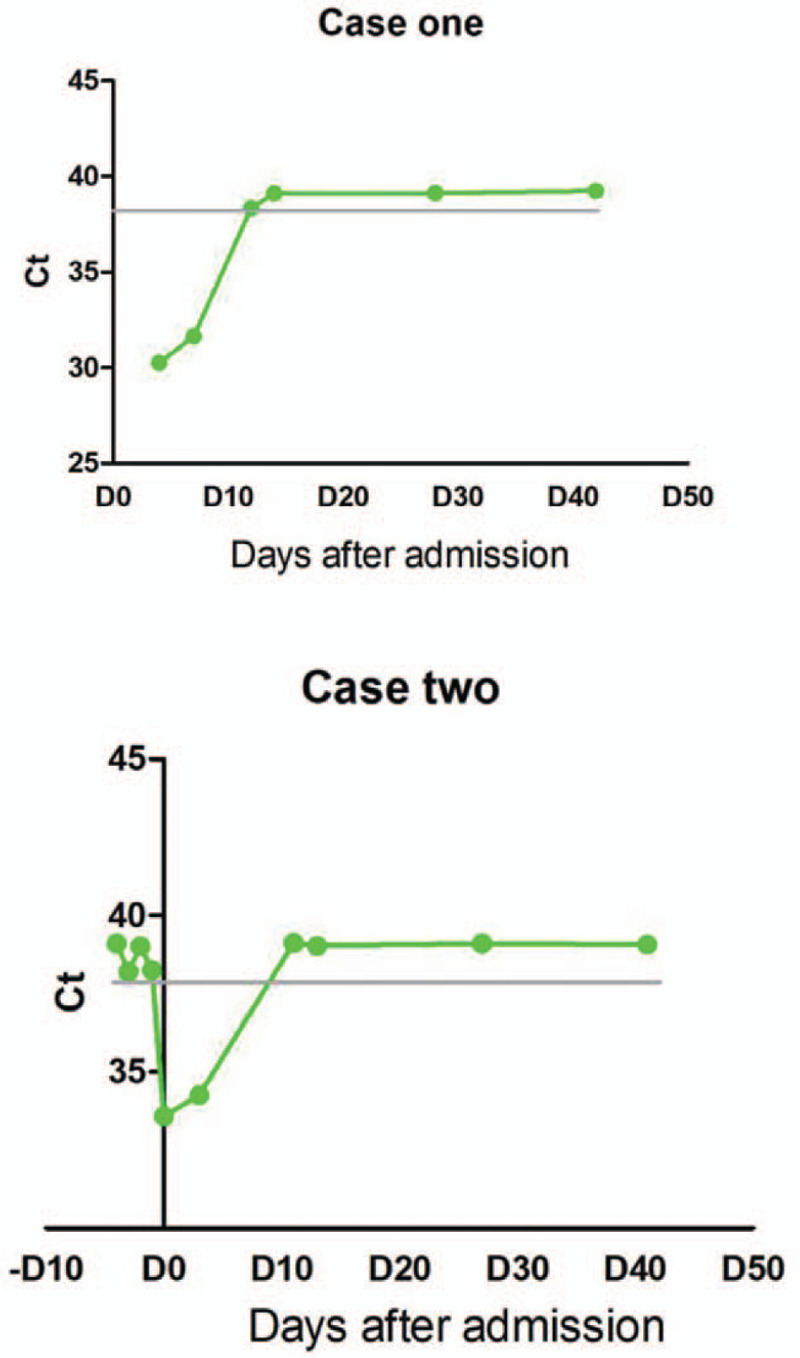
The changes in the qPCR cycle threshold (Ct) for SARS-CoV-2 in the 2 cases. The normal reference for the PCR Ct value ranges from 38 to 40. A Ct value of less than 38 is considered to be positive.

Based on national guidelines, the patient was discharged and remained in isolation for an additional 14 days. In the second and fourth weeks after discharge, routine blood and biochemistry tests showed no abnormalities, and the chest CT revealed that the lung lesions were completely absorbed. The patient was followed until April 30, 2020, during which time he did not exhibit fever, cough, diarrhea, fatigue, or other COVID-19 symptoms. Two other nasopharyngeal swabs were obtained on D28 and D42, respectively, and the results were negative for SARS-CoV-2 (Fig. 4).

The epidemiological survey for this patient revealed that he had 3 close contacts, including his wife, daughter, and son-in-law. None of these individuals developed any clinical signs of SARS-CoV-2 infection as of the last follow-up day for this patient.

### 2.2 Case 2

**Figure 5:**
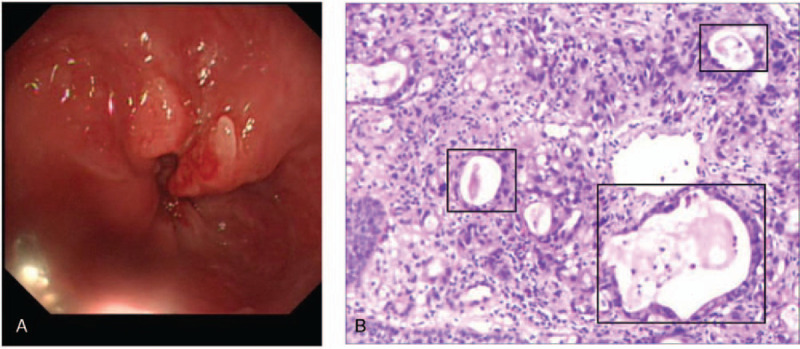
The cardia adenocarcinoma pathology of case 2. A. Electronic gastroscopy on August 15, 2019, showed an ulcer from the lower esophagus to the bottom of the cardia and stomach. B. Pathologic diagnosis was cardiac adenocarcinoma (medium low differentiation, luaren type: mixed type, significant × 100).

The second case was a 42-year-old male with a history of tuberculosis for more than fifteen years, which had been cured, and no other specific chronic diseases were noted. On September 17, 2019, a “lower esophagus and partial gastrectomy” was performed with a pathology diagnosis of cardia adenocarcinoma (middle to low differentiation, Luaren type: mixed) (Fig. 5). After 3 cycles of chemotherapy of “docetaxel + lobaplatin,” administered in Enshi, the fourth cycle of chemotherapy was given in Wuhan on January 10, 2020. Six days later, when the chemotherapy had ended, the patient was discharged and returned to Enshi.

Due to his Wuhan travel history, he received 4 consecutive nasopharyngeal swabs for PCR detection of COVID-19, all with negative results. However, the fifth swab was positive on February 22, 2020. The patient was admitted to the hospital that same day (D0). This patient did not exhibit fever, cough, diarrhea, fatigue, or other COVID-19 symptoms, and his routine blood and biochemistry tests remained normal. A lung CT scan showed no signs of viral infection. Therefore, this patient was diagnosed as an asymptomatic carrier of COVID-19. He remained in isolation in the hospital for 14 days and was discharged after 2 consecutive negative PCR tests for SARS-CoV-2 nucleic acid (D11, D13). The patient was followed until April 30, 2020, and he never exhibited fever, cough, diarrhea, fatigue, or other common COVID-19 symptoms. His routine blood and biochemistry tests and chest CT scans were normal in the second and fourth weeks after being discharged. The results of 2 additional nasopharynx swabs on D27 and D41 were negative for SARS-CoV-2 (Fig. 4).

The epidemiological survey for this patient showed that he had 5 close contacts (his wife, eldest son, second son, father-in-law, and mother-in-law). His father-in-law was diagnosed with COVID-19 pneumonia (moderate type) 12 days after the patient returned home from Wuhan. After treatment in a hospital designated for COVID-19 patients, the father-in-law was discharged, and follow-up tests were completed. Except for his father-in-law, no other close contact of this patient presented signs of SARS-CoV-2 infection as of the last follow-up day.

## 3 Discussion

Patients with cancer have a higher risk of adverse consequences or comorbidities due to their systemic immunosuppression caused by the malignancy as well as anticancer treatments, such as chemotherapy. Analysis of 2,007 cancer patients infected with COVID-19 from 575 hospitals in 31 provincial administrative regions revealed that patients with cancer exhibited higher risk and poorer outcomes than infected individuals without cancer.^[2]^ Also, patients who underwent chemotherapy or surgery within a month of becoming infected had a significantly higher risk of clinically severe adverse reactions than individuals who did not receive chemotherapy or surgery.^[2]^

Interestingly, the presence of some exceptions might indicate specific clues in how to evaluate COVID-19 patients with cancer better. A female COVID-19 pneumonia patient who had recently started chemotherapy for recurring breast cancer had a faster recovery than her spouse, who also was infected with COVID-19.^[3]^ The 2 patients in this study who experienced malignancy and were undergoing or just completed anticancer chemotherapy treatment presented favorable outcomes after being infected with COVID-19.

The 2 patients in this study were in immunocompromised states. The patient with cardia carcinoma (case 2) had just completed a course of chemotherapy in Wuhan, and the patient with hepatocellular carcinoma (case 1) was still undergoing chemotherapy. Their immunocompromised state likely enhanced their susceptibility to SARS-CoV-2. An immunocompromised state also occurs in transplant recipients who undergo long-term immunosuppression. During the onset of the COVID-19 epidemic, transplant recipients in China did not exhibit a higher infection rate compared to the general population due to clinicians’ awareness of the patients’ need for self-protection. However, cancer patients lack this awareness in general, even patients who had received recent anticancer treatments, such as the 2 cases in this report.

Our work with transplant recipients demonstrated that once the recipients were infected by SARS-CoV-2, while their clinical manifestations tended to be more severe, their outcomes were generally superior to those observed for severe cases who were not transplant recipients.^[4–6]^ The current patient with hepatocellular carcinoma exhibited similar clinical characteristics. He was diagnosed with a mild COVID19 infection after positive detection of COVID-19 by qPCR from a nasopharyngeal swab. The patient exhibited a mild fever, dry cough, and positive findings on a chest CT scan. By day 2 after admission, he had rapidly deteriorated into a critical state with bilateral ground-glass opacity observed in his lungs. He required invasive ventilation to maintain his blood oxygen levels. To our astonishment, 4 days later, he was able to maintain his oxygen saturation after the ventilator was withdrawn. The patient recovered and was discharged 15 days after admission. Even though cytokine levels were not assessed in this patient, we inferred that a cytokine storm did not occur, which might explain why this patient was able to recover from his critical state. An immunocompromised state is beneficial in that it can inhibit triggering and exacerbating a cytokine storm. In severe and critical patients with allo-transplants, we detected only low levels of IL-2 and IL-6 (data not shown). It is likely that the 2 cases in this report directly or indirectly benefited from their immunocompromised state (low lymphocytes) due to their recent chemotherapy. Other explanations for the recovery from critical status include rapid and sufficient medical support, oxygen supplementation, and no comorbidities.

When the patients were diagnosed with COVID-19, the qPCR cycle threshold (Ct) for the 2 patients was 30.25 and 33.58, respectively, indicating a relatively low viral load, which also could have contributed to their favorable outcomes. Furthermore, concerning the 8 close contacts of the combined 2 cases, only 1 was confirmed to have been infected with SARS-CoV-2. The low infection rate of 12.5% (1/8) also could be related to the low viral load of the 2 patients, in addition to the possibilities of different viral strains and mutations.^[7]^

One interesting question is, why were the viral loads relatively low in the 2 immunocompromised cases? Also, the duration of viral clearance (based on nasopharyngeal swab specimens) was short in both cases (5 and 8 days, respectively). As shown in the first reported case in the US, the oropharyngeal swab specimens revealed a viral clearance duration of 8 days (from day 4 to day 12).^[8]^ One explanation is that the recent chemotherapy damaged the general immune function but not the anti-SARS-CoV-2-specific immunity. Therefore, further analyses of lymphocyte subsets and specific functions are needed. Moreover, after the initial viral infection, no viral recurrence was detected in either patient throughout the follow-up period.

Whether or not asymptomatic carriers possess infectivity remains a concern. The current cases showed a 20% (1/5) infection rate. However, only an extensive epidemiological survey could clarify this question, which is currently underway in the cities of Enshi and Wuhan in Hubei Province, China. The surveys of viral infections and titers of sera-specific antibodies are ongoing.

The 2 cases in this report did not experience any recurrence of COVID-19 infection during the entire time their cases were followed. After they recovered, they returned to normal life and continued their medical consultation for their anticancer treatments.

In conclusion, as pointed out by Liang et al,^[2]^ cancer patients in general experience higher risk and poorer outcomes with regard to SARS-CoV-2 infections. However, current or recently completed anticancer treatment, especially chemotherapy, could have specific effects on immune function that result in unique outcomes due to individual variations. These outcomes may result in more favorable responses to COVID-19 infection in cancer patients. It also is likely that older cancer patients are likely to have worse adverse outcomes from COVID-19 infection. Both individuals discussed in this report were under the age of 60. In addition to age, the general condition of the patient, and the presence of little or no comorbidity, as well as prompt and sufficient medical support, substantially influences the outcome for the patient. Appropriate public health interventions and additional specific epidemiological surveys are needed to guide future strategies to improve the health of our communities.

Immunity is a 2-edged sword for COVID-19 evaluation and treatment. With a weak immune system, COVID-19 could develop quickly and cause the patient's health to become severely compromised. On the other hand, the possibility of a lethal cytokine storm could be reduced, resulting in an improved outcome. The current 2 cases received recent chemotherapy for neoplasias, especially case 1 who was continuing sorafenib treatment at the time of his COVID-19 infection. In fact, in immunocompromised COVID-19 cases such as transplant recipients, a more rapid clinical progression of the disease compared to the general population has been observed by us and others,^[4,9]^ which is consistent with the progression of the COVID-19 infection in case 1. It is generally accepted that the doses of immunosuppressants for transplant recipients with COVID-19 are reduced or withdrawn until recovery,^[4,9–11]^ even though the withdrawal of immunosuppressive therapy can be associated with exacerbation of the inflammatory response to viral infection.^[10]^ Therefore, the superior outcomes of these 2 cases and a COVID-19 patient with cancer reported in Switzerland^[3]^ could be partly explained by their recent chemotherapy, which impacted their immune status. Also, the relatively younger age and lack of comorbidities of these 2 patients were likely factors in their successful recovery from COVID-19.

Therefore, it is essential to examine additional cases of cancer patients infected with COVID-19 to determine whether current or recently ended treatment with antineoplastic drugs enhances a patient's ability to respond favorably to COVID-19 infection. Clinicians should note that recent anticancer treatment, especially chemotherapy, could have specific effects on immune function that result in unique outcomes due to individual variations, and they should propose guidelines for diagnosis or treatment that are specific for individual patients.

## Acknowledgments

The authors express their gratitude to EditSprings (https://www.editsprings.com/) for their expert linguistic services.

## Author contributions

**Funding acquisition:** Nianqiao Gong.

**Investigation:** Gang Lu, Jian Li, Yun Wu, Xiaofeng Wu, Yanjie Huang, Yi Ai, Dongfeng Xiang, Bo Zeng, Zuwang Zhang.

**Supervision:** Jun Yang.

**Writing –** original draft: Zhaoqing Ju.

**Writing –** review & editing: Nianqiao Gong.

## References

[1] Pan A Liu L Wang CL. Association of public health intervention with the epidemiology of the COVID-19 outbreak in Wuhan, China. JAMA 2020;323:1915–23. 32275295 10.1001/jama.2020.6130 PMC7149375

[2] Liang WH Guan WJ Chen RC. Cancer patients in SARS-CoV-2 infection: a nationwide analysis in China. Lancet Oncol 2020;21:335–7. 32066541 10.1016/S1470-2045(20)30096-6 PMC7159000

[3] Spezzani V Piunno A Iselin HU. Benign COVID-19 in an immunocompromised cancer patient - the case of a married couple. Swiss Med Wkly 2020;150:w20246. 32277836 10.4414/smw.2020.20246

[4] Zhu L Gong NQ Liu B. Coronavirus disease 2019 pneumonia in immunosuppressed renal transplant recipients: a summary of 10 confirmed cases in Wuhan, China. Eur Urol 2020;77:748–54. 32317180 10.1016/j.eururo.2020.03.039 PMC7166037

[5] Xia L Changsheng M Zhixiang W. Clinical analysis of 2019 coronavirus disease (COVID-19) on one case with living-related kidney transplantation. Chin J Organ Transplant 2020;3:149–53.

[6] Chen S Yin Q Shi H. A familial cluster, including a kidney transplant recipient, of coronavirus disease 2019 (COVID-19) in Wuhan, China. Am J Transplant 2020;20:1869–74. 32243690 10.1111/ajt.15903 PMC9800498

[7] Qu JX Zhou J Zhang J. RBD mutations from circulating SARS-CoV-2 strains enhance the structure stability and infectivity of the spike protein. BioRxiv 2020.

[8] Holshue ML DeBolt C Lindquist S. Washington state 2019 nCoV case investigation team. First case of 2019 novel coronavirus in the United States. N Engl J Med 2020;382:929–36. 32004427 10.1056/NEJMoa2001191 PMC7092802

[9] Akalin E Azzi Y Bartash R. Covid-19 and kidney transplantation. N Engl J Med 2020;382:2475–7. 32329975 10.1056/NEJMc2011117 PMC7200055

[10] Nair V Jandovitz N Hirsch JS. COVID-19 in kidney transplant recipients. Am J Transplant 2020;20:1819–25. 32351040 10.1111/ajt.15967 PMC7267603

[11] Guillen E Pineiro GJ Revuelta I. Case report of COVID-19 in a kidney transplant recipient: does immunosuppression alter the clinical presentation? Am J Transplant 2020;20:1875–9. 32198834 10.1111/ajt.15874 PMC7228209

